# Digital learning of clinical skills and its impact on medical students’ academic performance: a systematic review

**DOI:** 10.1186/s12909-024-06471-2

**Published:** 2024-12-18

**Authors:** Richard G. McGee, Stuart Wark, Felista Mwangi, Aaron Drovandi, Faith Alele, Bunmi S. Malau-Aduli, The ACHIEVE Collaboration

**Affiliations:** 1 School of Medicine and Public Health, College of Health, Medicine and Wellbeing, University of Newcastle, Newcastle, NSW 2308 Australia; 2 Campbelltown Hospital, South Western Sydney Local Health District, Campbelltown, NSW Australia; 3 School of Medicine, Western Sydney University, Campbelltown, NSW Australia; 4 School of Rural Medicine, Faculty of Medicine and Health, University of New England, Armidale, NSW Australia; 5 School of Medical Sciences, Faculty of Biology, Medicine and Health, University of Manchester, Manchester, Greater Manchester, UK; 6 School of Health, University of the Sunshine Coast, Sunshine Coast, QLD Australia; 7 Academy for Collaborative Health Interprofessional Education and Vibrant Excellence, University of Newcastle, Newcastle, NSW Australia

**Keywords:** Clinical skills, Medical education, Pedagogy, Digital learning

## Abstract

**Background:**

The constraints imposed by the COVID-19 pandemic has led to the rapid development and implementation of digital methods for teaching clinical skills in medical education. This systematic review presents both the benefits, challenges, and effectiveness of this transition.

**Methods:**

A systematic search of six electronic databases (SCOPUS, Medline, CINAHL, PsycINFO, ERIC & Informit) was conducted on 1st October 2023 and updated on 1st April 2024 to identify peer- reviewed articles, from 2019 onwards, which used any type of digital tool (online or otherwise) to teach clinical skills to medical trainees (undergraduate or postgraduate) and were published in English language. The primary outcome synthesised was the reported effectiveness of these digital tools in the development of clinical skills. Risk of bias of included studies was assessed using the Quality Assessment With Diverse Studies (QuADS) tool.

**Results:**

Twenty-seven studies involving 3,895 participants were eligible for inclusion in this review. The QuADS quality assessment scores ranged from 22 to 35, indicating medium quality and thirteen of the studies were randomized trials. Overall, digital teaching of clinical skills demonstrated improved or comparable outcomes to in-person teaching. There was a beneficial effect of digital learning tools on assessment outcomes, with meta-analysis showing a mean difference increase of 1.93 (95% CI 1.22 to 2.64), albeit with a high amount of statistical heterogeneity I2 97%, *P* < 0.001. Digital clinical skills teaching also resulted in improved student satisfaction scores in many situations and was also shown in one study to be cost effective.

**Conclusion:**

Teaching of clinical skills using digital tools is an important alternative to the traditional format of face-to-face delivery, which is resource intensive and difficult to implement during a pandemic. This review demonstrates their potential efficacy in improving education outcomes, student satisfaction and potentially reducing costs. However, the integration of traditional and innovative digital teaching methods appeared to provide the most comprehensive learning experience. Future research could focus on longitudinal studies to assess the long-term impact and efficacy of different digital and blended learning modalities on the acquisition of clinical skills and professional competencies.

**Supplementary Information:**

The online version contains supplementary material available at 10.1186/s12909-024-06471-2.

## Background

The COVID-19 pandemic precipitated a paradigm shift in medical education, necessitating a global re-evaluation of pedagogical strategies to sustain the progression of clinical skills acquisition, which is vital for patient-centred care [1–3]. Historically, the foundation of medical training has been the integration of theoretical knowledge with practical, experiential learning, achieved through direct patient interactions and simulated clinical scenarios [4]. This model is especially crucial for students navigating the transition from theoretical pre-clinical studies to the hands-on clinical environment, ensuring they acquire essential skills such as history-taking, physical examination techniques, and procedural competencies requisite for effective patient care and hospital placements [5].

Prior to COVID-19, some universities used digital learning for various reasons, including increased accessibility and user preference. The advent of the COVID-19 pandemic, accompanied by stringent physical distancing mandates and lockdowns, significantly disrupted traditional clinical education pathways. In response, numerous educational institutions swiftly transitioned to remote and digital platforms for clinical training [2, 6–8]. This shift towards online clinical skills learning leverages electronic technologies to foster clinical reasoning, communication skills, and other core medical competencies, employing digital media to deliver consistent educational content, introduce innovative instructional methods, and facilitate the documentation of student engagement and performance assessments [9]. Nonetheless, the transition introduces several challenges, including diminished practical exercise opportunities, constraints on teaching physical examination techniques, and hurdles in the comprehensive evaluation of clinical competencies [4]. These challenges have spurred concerns about the efficacy of online and blended learning models in adequately preparing students for their clinical roles [4].

As the landscape of medical education continues to adapt to the challenges posed by the pandemic, the experiences and lessons learned from this period of enforced pedagogical innovation are poised to shape future educational strategies. Although most education has shifted back to in-person learning, there are some adaptations that may improve traditional teaching. The primary goal remains to ensure that all students attain the necessary clinical competencies, regardless of the educational formats employed. This period of accelerated adaptation may herald the development of more robust and flexible teaching methodologies, enhancing the acquisition and refinement of clinical skills in preparation for the demands of post-pandemic healthcare environments [10].

Given the evolving nature of medical education in response to the challenges posed by the pandemic, it is crucial to systematically review the experiences and lessons learned during this period of enforced pedagogical innovation. Previous systematic reviews in this area predate the COVID-19 pandemic [11, 12]. The current dearth of recent literature in this area necessitates a systematic review on this topic. Therefore, this systematic review aims to examine the impact of digital and blended learning environments on medical students’ academic performance in clinical skills training following the COVID-19 pandemic. We also aim to evaluate the effectiveness of educational adaptations, specifically digital media tools such as online modules, instructional videos, and lecture recordings, in maintaining high standards of clinical skills education.

## Methods

This systematic review was reported according to the Preferred Reporting Items for Systematic Reviews and Meta-Analyses (PRISMA) guidelines [13].

### Search strategy

Six electronic databases comprising MEDLINE, SCOPUS, PsychInfo, CINAHL, ERIC, and Informit were searched on 1st October 2023 and the search was updated on 1st April 2024. Reference lists of included studies and relevant systematic reviews were also searched to identify other eligible studies not captured by the search strategy. Search terms used related to the concepts of clinical skills, clinical competency, medical education, and online learning. The full search strategies for each database are outlined in **Supplement 1**. Search results were imported into Covidence systematic review software (Veritas Health Innovation, Melbourne, Australia. Available at www.covidence.org) for screening.

### Eligibility criteria

To be eligible for inclusion, studies had to involve Population: medical trainees (either undergraduate or postgraduate) where Intervention: any type of digital media or tool (online or otherwise) was used to teach clinical skills compared to any other teaching format, and academic performance was measured or assessed. Studies involving other healthcare professions, such as chiropractic students, residents, resident physicians, or allied health students, were excluded. Digital media included electronic resources, platforms, applications, and other interactive digital media that used digital technology to create, deliver, or manage educational content, for example instructional videos, lecture recordings, and other digital resources used to facilitate learning were eligible for inclusion. Virtual reality (VR) technologies were not included in our systematic review because they typically require face-to-face delivery and specialised equipment, which differs from the digital media tools we aimed to evaluate. Our review focused on digital learning modalities that are accessible remotely and can be used by students without the need for physical presence or specialized hardware. Mixed interventions, e.g. both face-to-face and digital components, were also included. Clinical skills included competencies such as history-taking, physical examination, communication skills, and clinical reasoning. Procedural skills and technical competencies were included if they involved direct patient care. Clinical skills learning was defined as development of clinical reasoning, communication skills, and other medical competencies e.g. professionalism, ethical decision-making, teamwork, cultural competence, and evidence-based practice. Studies without a Comparison group were excluded. As the focus of the review was on COVID-19 pandemic’s effect, the search was limited to studies conducted from 2019 onwards. Additionally, included studies had to be peer-reviewed journal articles published in the English language. Commentaries and studies that only reported the Outcome of satisfaction levels (i.e. where academic performance was not assessed) were excluded. We defined assessment of academic performance as measuring a student’s competence in applying theoretical knowledge to practical clinical tasks [14].

### Data extraction

Using COVIDENCE systematic review software, two authors independently screened studies for inclusion. Any discrepancies were resolved by a third author. After the studies were selected, a data extraction template was used to extract required information. Extracted variables included study authors, year published, number of participants, as well as review-specific outcomes such as type of digital teaching tool used, clinical skills taught, and academic performance measures.

### Data analysis and synthesis

Numerical data was reported as mean and standard deviation (SD) or median and interquartile range, and categorical data as number and percentage. Meta-analyses on studies were conducted using Review Manager 5 software (Cochrane collaboration). The means and standard deviations from each trial were identified for inclusion in the meta-analysis. We used the Mantel-Haenszel random‐effects model to account for potential variability in participant conditions between studies and to calculate the pooled estimates (mean difference) and 95% confidence intervals. We assessed for apparent inconsistency in our results by examining methodological and statistical heterogeneity. We evaluated methodological heterogeneity by considering similarities amongst the included studies in terms of study design, participants, interventions, and outcomes, and used the data collected from the full‐text reports. We assessed statistical heterogeneity by calculating the ChI2 test or I2 statistic, judging an I2 value of 50% and a Chi2 P value of 0.05 or less as indicating substantial statistical heterogeneity. For thematic analysis we read each study in depth to identify study variables. Two investigators independently coded the data, and discrepancies were resolved through discussion and consensus. A third reviewer was consulted when necessary to resolve any remaining differences. Extracted data was summarised narratively, taking into consideration the interventions reported, and primary and secondary outcomes relevant to clinical skills teaching of medical students.

### Risk of bias

Risk of bias was assessed using the ‘Quality Assessment with Diverse Studies’ (QuADS) tool [15]. Two authors independently assessed risk of bias using the QuADS tool, reaching consensus on final scores through discussion. Discrepancies were resolved by a third reviewer. The QuADS tool was deemed suitable as it allows for assessment of a broad range of methodologies and it has been reported as having good inter-rater reliability and validity [15]. The tool has 13 criteria to assess study quality, each of which are scored between zero (not stated at all) and three (explicitly described) (see Supplement 2). To ensure consistency in the assessment of quality, for each reviewed paper, the 13 criteria scores were summed and expressed as a percentage of the maximum possible score. This approach allowed for comparison of quality across the different papers. Interpretation of the quality evidence involved classification of total scores into low (< 60%), medium (60–80%) or high (> 80%). Studies were not excluded based on their quality rating, though the significance of their findings were considered when reporting the results and drawing conclusions based on the findings of all the included studies.

## Results

### Search results and study characteristics

**Fig. 1:**
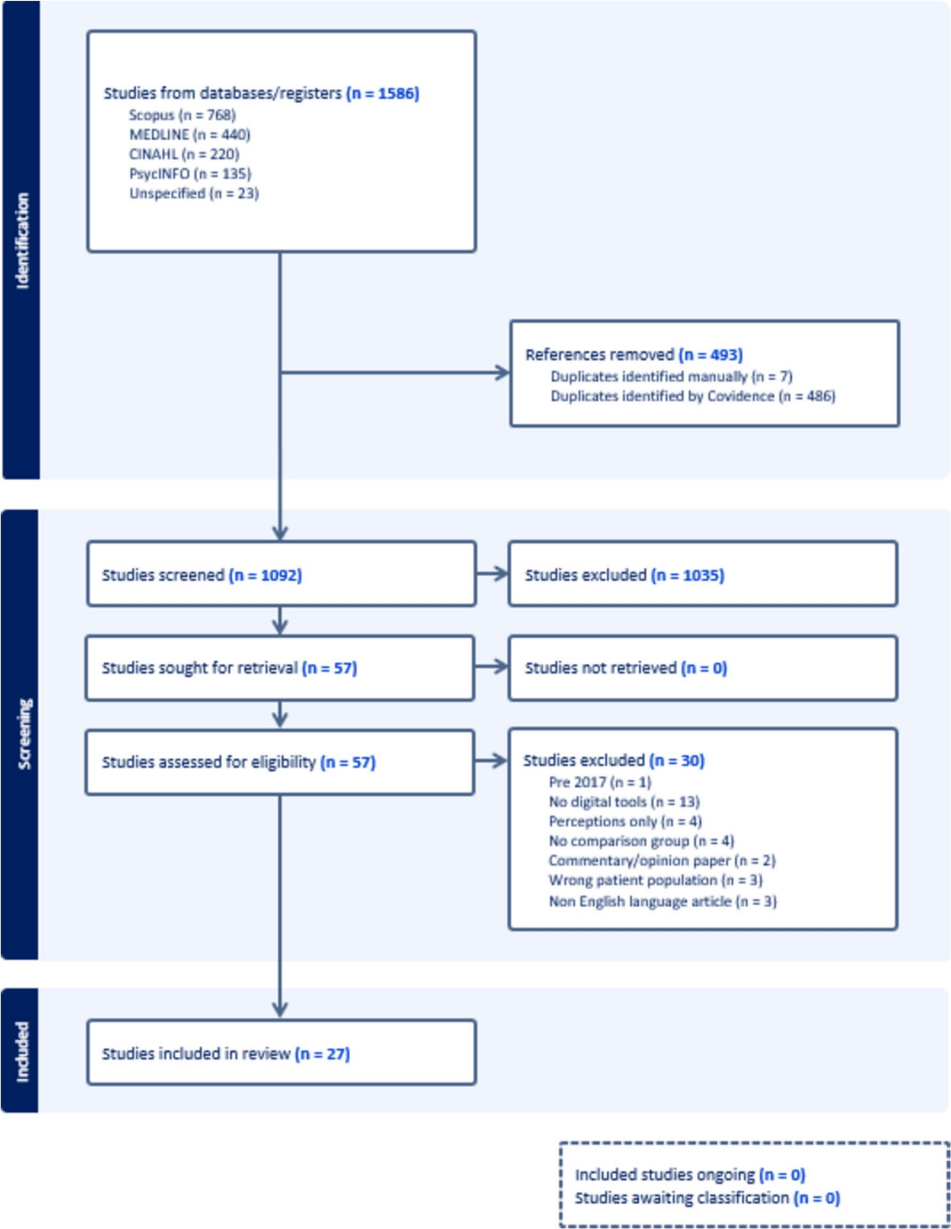
PRISMA flow diagram of study screening and selection

Of 1092 unique records identified from the search strategy, 1035 (94.8%) were excluded through title and abstract screening, leaving 57 for full-text review. Of these, 30 were excluded for a variety of reasons, see Fig. 1 for details. Twenty-seven studies which involved 3,895 participants were included in the final analysis [16–42]. Figure 1 illustrates the screening and study selection process.

The studies were published from a wide range of countries, five from the United Kingdom (UK) [17, 27, 30, 35, 37]; four from Germany [20–22, 36]; and two from the United States of America [23, 26]. Of the studies, twelve were randomized trials [17, 18, 20–22, 25, 27, 30–32, 35, 36]; four were prospective observational studies [23, 24, 28, 34]; four were mixed methods studies [29, 39, 41, 42] and the remainder were a variety of study designs. The interventions were also varied, the most common formats used were videos (*n* = 12) and online modules/ courses (*n* = 6). Table 1 summarises the characteristics of the included studies.

**Table 1:** Characteristics and outcomes of included studies

Study (Year)	Country	Study type	Participants (Sample size; sex)	Mean age in years ± SD	Type of intervention	Type of clinical skills assessed	Summary of findings
Durán-Guerrero (2019) [16]	Colombia	Quasi-experimental and retrospective study	Medical students (294; NS)	NS	Five online radiology education modules	Diagnostic imaging knowledge	The online modules improved the median final exam score compared to the traditional lecture learning method only. There was a significant association between gained knowledge and the number of visits to online modules.
Nathan (2022) [17]	UK	Randomised controlled trial	Medical students from all year groups (72; 65% female)	21.3 ± 2.1	Virtual classroom basic surgical skills training	Proficiency in placing three interrupted sutures with hand-tied knots	Suturing and knot-tying proficiency did not differ between virtual classroom training and face-to-face training. Virtual classroom training and face-to-face training produced superior results compared to computer-based learning. The costs per attendee associated with virtual classroom training, face-to-face training and computer-based learning were £22.15, £39.69 and £16.33, respectively.
Cho (2022) [18]	South Korea	Randomised controlled trial	Third-year medical students (68; 38.2% female, 61.8% male)	24 (range; 23–25)	Computer-based learning using non-interactive instructional video	Cardiopulmonary resuscitation	Although all students in both groups passed the post-training assessment, the computer-based learning group demonstrated less superior understanding during CPR, including fewer calling for assistance and using of defibrillator compared to the face-to- face group.
Azman (2021) [19]	Malaysia	Prospective interventional study	Final year medical undergraduates (45; (58% female, 42% male)	24 (*n* = 31) 25 (*n* = 13) 26 (*n* = 1)	Virtual classroom otoscopy training	Level of confidence and diagnostic ability in common ear pathologies	Level of confidence and diagnostic ability scores were significantly higher after the intervention and four months following the intervention. Both level of confidence and diagnostic ability scores showed sustained improvements at four months post intervention across.
Grosser (2019) [20]	Germany	Randomised controlled trial	Medical student users from the TSC-database (114; 51% female)	24.3 ± 7.38	Videos about Anterior Cruciate Ligament reconstruction	Knowledge	Participants in the video-based format acquired more clinical knowledge than in the lecture condition but there was no difference in acquisition of anatomical knowledge. Participants perceived the video-based format to be superior in comprehensibility of the presentation, conceivability of the surgical procedure and to be more entertaining.
Lehmann (2019) [21]	Germany	Randomised controlled trial	5th-year medical students (103; 40% female, 60% male)	25.1 ± 2.62	Interactivity and animations in virtual patients	Paediatric basic life support skill	The intervention did lead to a difference in the adherence to the correct algorithm. Self-instruction with animated media – through videos or animation-enriched virtual patients – resulted in a better adherence to temporal demands compared to training with static virtual patients. The static virtual patients group performed considerably compared to the animated media and animation-enriched virtual patients.
Herrmann-Werner (2019) [22]	Germany	Randomised controlled trial	Medical students (46; 74% female)	25.4 ± 2.3	Videos on patient communication	Empathy and competency	The videos significantly improved the students’ competency in dealing with e-patients as judged by expert video raters. Students’ rating showed a similar non-significant trend.
Berland (2019) [23]	United States	Prospective study	First-year matriculating students in year one, year two and year three in a medical school (234; NS)	NS	Online-training modules on opioid overdose prevention	Knowledge, attitudes and preparedness	There were statistical differences that the authors deemed as not meaningful in knowledge, attitudes and preparedness.
Hansen (2020) [24]	Denmark	Prospective case comparison study	Clerkship students (128; 70% female)	26.3	Videos on Mental Status Examination (MSE)	Mental Status Examination	The students with video access scored higher compared to students without access.
Nazari (2020) [25]	Netherlands	Randomised controlled trial	Medical students (43; 51% female, 49% male)	20 [19-21] (median [IQR])	Step-by-step or continuous video-demonstration	Open inguinal hernia repair	The surgical performance was not significantly different between both groups. The step-by-step group perceived a lower extraneous cognitive load compared to the continuous group.
Power (2020) [26]	United States	Pretest-post-test study	Second-year medical students (147; NS)	NS	Online clinical vignette, photo, and auditory clips	Cardiac auscultation skills	The cardiac auscultation skills score was higher post-intervention compared to the preintervention score.
Plackett (2020) [27]	UK	Randomised controlled trial	Final year undergraduate medical students (264; 46% female, 54% male)	20–22 (*n* = 5) 23–24 (*n* = 152) 25–26 (*n* = 68) 27–28 (*n* = 21) > 29 (*n* = 18)	eCREST — the electronic Clinical Reasoning Educational Simulation Tool.	Clinical reasoning, knowledge and diagnostic choice	eCREST improved students’ ability to gather essential information from patients compared to the control group. Most students in the intervention group agreed that eCREST helped them to learn clinical reasoning skills.
Viljoen (2020) [28]	South Africa	Prospective study	Fourth-year medical students (153; NS)	NS	Web application	Electrocardiogram competence	Blended learning with the web application was associated with significantly better scores compared to conventional teaching in immediate and delayed post-intervention tests. The blended learning was associated with better confidence in electrocardiogram analysis and interpretation.
Kasai (2021) [29]	Japan	Mixed methods	Fifth-year medical students (43; NS)	NS	Simulated electronic health records, electronic problem-based learning and online virtual medical interviews	Multiple clinical skills: Medical interviewing, physical examination, professionalism, clinical judgement, counselling, organisation/efficiency and documentation	Using simulated electronic health records resulted in significant improvement in writing daily medical records and medical summaries. Students using electronic problem-based learning and online- virtual medical interviews reported significant improvement in medical interviews and counselling. Students indicated that clinical clerkships were more useful for learning medical interviews, physical examinations, and humanistic qualities than the online education for clinical practice.
Brewer (2021) [30]	UK	Randomised controlled trial	Pre-clinical medical students (67; 55% female, 45% male)	21 ± 2.03	Online video	Examination of the shoulder joint	Mean post-intervention scores were highest in face-to-face group followed by video and textbook groups, respectively. There was no score change from day 5 to day 19 post-intervention.
Vincent (2022) [31]	Switzerland	Randomised controlled trial	Fourth-year medical students (160; 88 (55.0%) female)	22.8 ± 4.2	Videos	Breaking bad news skills	The number of correctly identified breaking bad news elements did not differ between control and intervention group, but the mean number of inappropriate breaking bad news elements was significantly lower in the intervention than in the control group.
Zaghal (2022) [32]	Lebanon	Randomised controlled trial	Pre-medical, first, and second-year medical students (118; 51% female, 49% male)	21.4 (range; 18–27)	Interactive tele-simulation sessions utilizing web-based video-conferencing technology and demonstration videos	Suturing	All participants were successful in placing three interrupted sutures, with no significant difference in the performance between the face-to-face and intervention groups. 25 (44.6%) of the respondents in the intervention group provided negative comments related to the difficulties of remotely learning visuospatial concepts.
Enoch (2022) [33]	South Africa	Non-random cross-sectional quasi-experimental study	Third-year medical students (488; 52% female, 48% male)	18–25 (*n* = 457) > 25 (*n* = 31)	Virtual simulation-based training using Zoom as the online platform	Affective, cognitive and psychomotor skills	The blended group had the highest scored followed by the e-learning group. The face-to-face group had the lowest score.
Huang (2022) [34]	China	Prospective and comparative stud	Fourth- and fifth-year medical students (76; 59.2% female, 40.8% male)	20.95 ± 0.67	Online course	Competency in the basic ocular examination	Students in the intervention group obtained overall higher scores in the slit lamp practical skills. The online course was deemed to increase learning interests and motivation but was preferred as an additional tool to traditional teaching methods rather than a replacement.
Flatt (2023) [35]	UK	Randomised controlled trial	Medical students (42; 54.8% female, 45.2% male)	20.5	Videos	Clinical examination of the shoulder joint	The intervention led to a significantly higher improvement in score.
Lang (2023) [36]	Germany	Randomised controlled trial	Medical students (55; 53% female, 47% male)	24.1 ± 3.5	Videos	Laparoscopic knot tying	The number of knot tying attempts until proficiency was reached did not differ between the intervention and control groups. However, there was a higher fraction of knots achieving technical proficiency in the intervention group after the first use of the coping model. The proportion of blinded attempts that met the criteria for technical proficiency was significantly higher for the intervention group.
Rajendran (2021) [37]	UK	Pretest-posttest design	Medical students (117; NS)	NS	Radiographs on an online user interface	Interpretation of chest radiographs	The high drop-out rate during the study that made the quantitative measurement of effectiveness difficult.
Gong (2021) [38]	China	Quasi-experimental study	Clinical medical undergraduates (200; 49.5% female, 50.5% male)	22 ± 0.37	Website with micro-lectures, demonstration videos, online exercises, screen-based simulation of clinical skills and a student-teacher communication platform	Cardiopulmonary resuscitation, pelvic examination, physical examination of children, urethral catheterisation and lumbar puncture	The results of the theoretical and practical assessments were higher in the intervention than in the control group. Blended learning was more effective for acquiring relevant knowledge, enhancing student-centered learning and improving clinical practice.
Saeed (2023) [39]	Pakistan	Mixed methods	First and second-year medical students (200; 53% male)	NS	Virtual platform (Microsoft Teams)	History taking, examination, basic life support skills	The OSCE scores showed significant improvement in two out of four repeated stations (abdominal and precordial examination). The questionnaires showed a significant improvement in seven of the nine skills taught. Session evaluations showed that most students were satisfied with the learning experience.
Heriwardito (2023) [40]	Indonesia	Randomised controlled trial	Second-year medical students (229; 59% female, 41% male)	NS	Videos	Endotracheal intubation and mask ventilation procedural skills	The rubric scores, global rating scores and the pass rate did not differ between the intervention and control groups.
Somera (2021) [41]	Brazil	Mixed methods	First-year medical students (189; 41.8% female, 58.2% male)	20.1 ± 2.6	Virtual microscopy	Pelvis histology knowledge	Virtual microscopy led to higher scores in subjective impressions such as handling, suitability, learning effectiveness and pleasure using the tools compared to optical microscopy. No statistically significant differences in academic performance were found between groups.
Saeed (2023) [42]	Pakistan	Mixed methods	Fourth-year medical students (200; 56% male)	NS	Hybridized video-based learning with simulation	Examination skills	Hybridization of video-based learning with simulation significantly improved self-efficacy scores for all examinations (cardiovascular, respiratory, neurological, and abdomen) and OSCE scores of the neurological and abdominal stations. The students stated that the intervention allowed reinforcement of basic concepts, retention, and further insight into clinical applications.

*NS* not specified, *IQR* interquartile range, *SD* standard deviation

### Effectiveness of digital learning tools

**Fig. 2:**
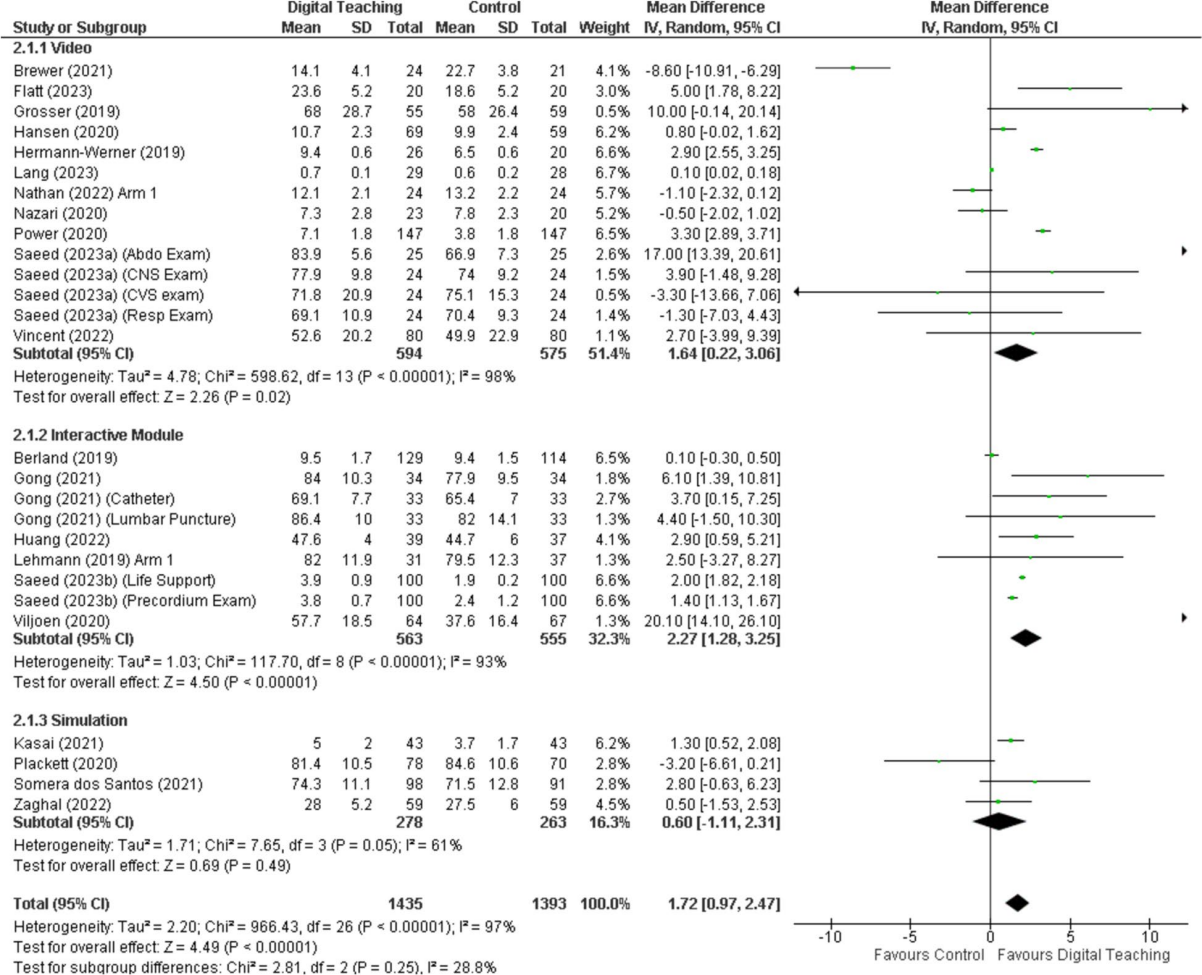
Forest plot of digital learning tools effectiveness by intervention

The included studies demonstrated that online and digital learning interventions could enhance acquisition and enhancement of clinical knowledge and skills across various domains, see Fig. 2. Online modules and videos significantly improved clinical knowledge and specific skill sets such as diagnostic imaging and cardiac auscultation [16, 18, 24, 26]. Video-based learning emerged as a particularly effective tool in enhancing clinical knowledge, though it did not significantly impact anatomical knowledge acquisition. Its perceived advantages in presentation comprehensibility and engagement highlight the potential for multimedia resources to enrich the learning experience [20]. Similarly, animated media and video-based interventions showed improved adherence to correct algorithms and competency in handling e-patient scenarios, demonstrating the effectiveness of dynamic visual content in medical education [21, 22]. Overall video tools showed a beneficial effect with a mean difference of 1.64 (95% CI 0.22 to 3.06), while interactive modules showed a beneficial effect with a mean difference of 2.27 (95% CI 1.28 to 3.25). These results are summarised in Fig. 2, which also demonstrates a high amount of statistical heterogeneity in the results.

**Fig. 3:**
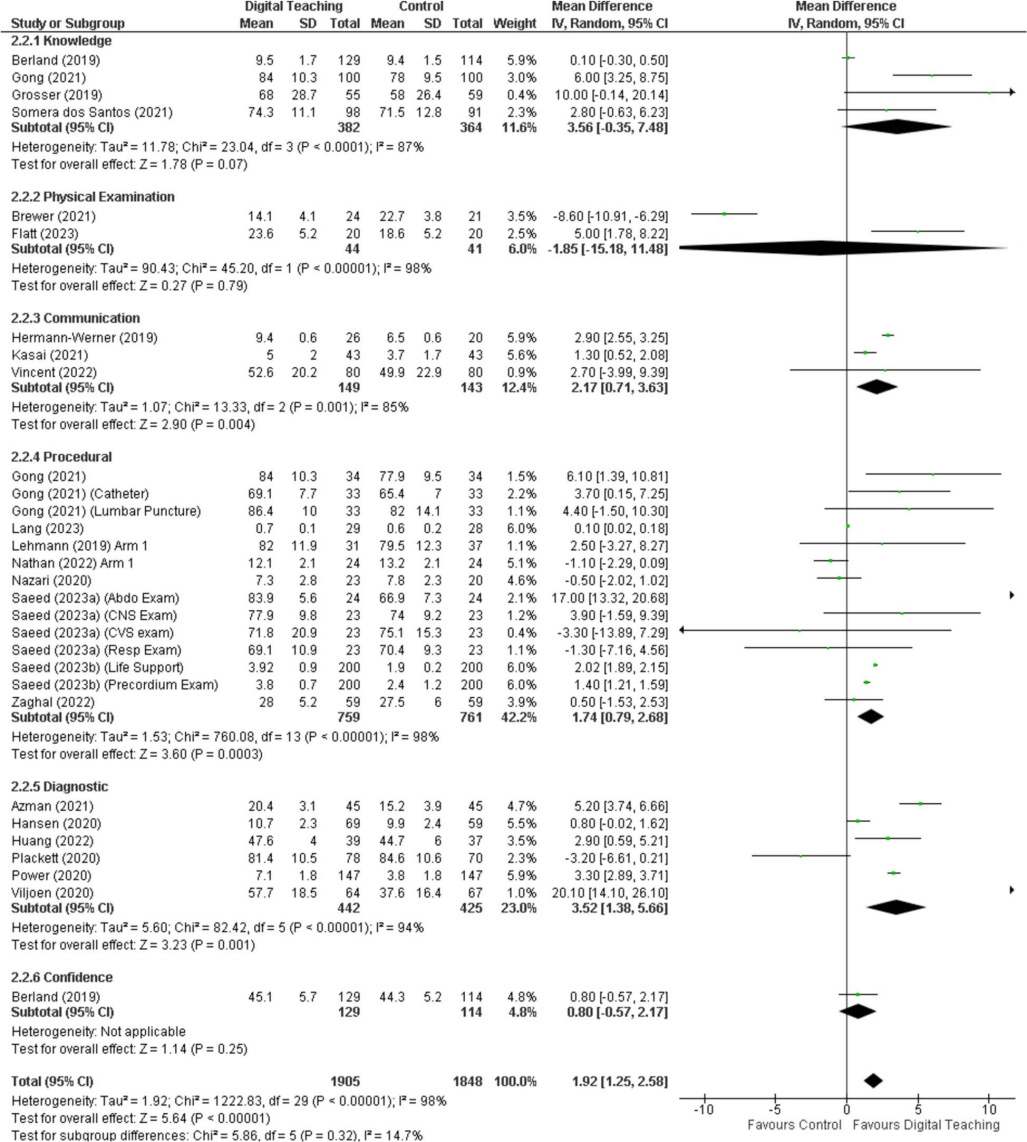
Forest plot by of digital learning tools effectiveness by skill area

Studies also explored the impact of learning modalities on specific skills such as cardiac auscultation and practical skills like suturing, finding that while certain interventions led to improvements in proficiency and confidence, the overall effectiveness varied across different competencies and learning outcomes [26, 36]. High dropout rates in some studies, posed challenges in accurately measuring the effectiveness of the interventions [37]. Plackett [27] reported that ‘eCREST’, an electronic clinical reasoning simulation tool, improved students’ ability to gather essential patient information and learn clinical reasoning skills. Comparatively, suturing and knot-tying proficiencies were found to be comparable across virtual classroom training and traditional face-to-face training, suggesting that certain practical skills can be effectively taught through virtual methods [17, 32]. However, both these training modalities were superior to computer-based learning in producing better outcomes, albeit with varying costs per attendee, highlighting the economic considerations in choosing the optimal training approach [17].In the area of CPR training, computer-based learning groups demonstrated a less comprehensive understanding of procedures such as calling for assistance and using a defibrillator, when compared to their counterparts in face-to-face training sessions [18]. This points towards the limitations of computer-based learning in fostering practical critical skills in emergency scenarios. Figure 3 summarises the effect of digital learning tools by skill area and shows a high amount of statistical heterogeneity in the results.

### Student performance and knowledge retention

Studies consistently showed that digital interventions positively affect student performance and knowledge retention. Confidence levels and diagnostic abilities significantly increased following interventions and remained elevated four months post-intervention in some studies, indicating sustainable improvements in these crucial areas [19]. Videos and animated media enhanced the comprehension and procedural adherence in clinical skills, indicating superior performance compared to traditional methods [20–22]. Studies using online modules or courses demonstrated an improvement in median final exam scores when compared to traditional lecture-based learning methods alone [16]. This is further supported by evidence showing a significant correlation between the frequency of visits to online modules and the extent of knowledge gained, indicating the value of engagement with digital resources.

Gong et al. [38] found that a blended learning platform enhanced student-centred learning and clinical practice, demonstrating higher theoretical and practical assessment scores. Blended learning approaches, incorporating web applications and simulated electronic health records, were associated with better immediate and delayed post-intervention test scores, enhanced confidence in electrocardiogram analysis, and improved clinical practice skills [28, 31]. This suggests that integrating online resources with traditional teaching methods can elevate learning outcomes significantly. Moreover, virtual microscopy was favoured over optical microscopy for its higher scores in subjective impressions, indicating a preference for digital tools in certain areas of study [41]. However, no significant differences were observed in academic performance between different groups [41].

Kasai et al. [29], highlighted that simulated electronic health records and online problem-based learning improved multiple clinical skills, including medical interviewing and counselling; while Huang et al. [34] reported that an online course enhanced competency in basic ocular examination, though students preferred using it as an additional tool rather than a replacement for traditional methods. Saeed et al. [39] and Vincent et al. [31] highlighted improvements in examination skills and breaking bad news skills, with significantly improved self-efficacy and OSCE scores, showcasing the effectiveness of hybridised video-based learning.

**Fig. 4:**
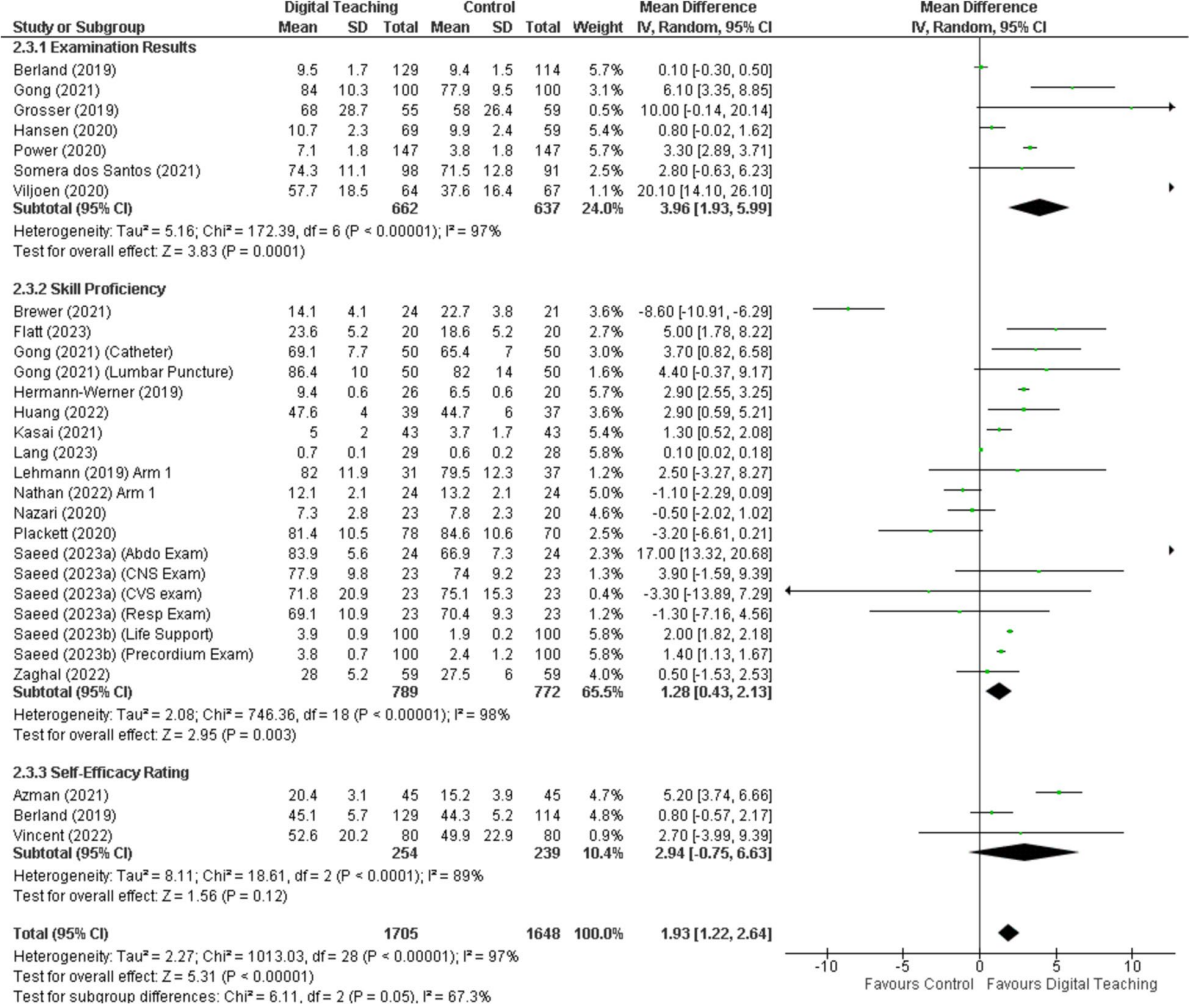
Forest plot by of digital learning tools effectiveness by outcome

Figure 4 summarises the effect of digital learning tools on assessment outcomes. The mean difference favours digital teaching, mean difference 1.93 (95% CI 1.22 to 2.64), with a high amount of statistical heterogeneity I2 97%, *P* < 0.001.

### Student satisfaction and engagement

Despite mixed efficacy for improving clinical skills there was a consensus of better engagement and satisfaction levels with digital clinical skills teaching. This increase in satisfaction did not appear specific to any one modality. For example, digital modules [34], videos [20, 25], web applications [28, 29], and even virtual meeting platforms (Microsoft Teams) [39, 42] all showed increased satisfaction scores by students. Nazari et al. [25] reported that step-by-step video demonstrations were perceived to have lower cognitive load and were preferred over continuous video demonstrations. Saeed et al. [39, 42] indicated that students were generally satisfied with their learning experience. Somera et al. [41] noted positive subjective impressions and engagement with blended learning and virtual microscopy environments. Only one study showed reduced satisfaction from students when it was described that learning visuospatial concepts remotely online was less optimal than in person training [32]. Unit of analysis issues precluded the use of proportional meta-analysis to create a pooled, overall proportion.

### Cost effectiveness of digital learning tools

The review also highlighted the potential cost-effectiveness of online learning modalities and noted improvements in long-term retention and confidence among learners. In 2022, Nathan et al. showed that computer based learning and virtual classroom training for teaching suturing skills resulted in a cost saving of 58.9% and 44.2% respectively compared to face-to-face training [17]. The other included studies did not include detailed cost breakdowns.

### Risk of bias of included studies

The QuADS results indicated that the scores ranged from 56 to 90%. There were more medium quality studies (*n* = 14) compared to low (*n* = 2) and high-quality studies (*n* = 12). Only two studies scored below 60%. Most studies had very low scores on stakeholder engagement in the research. The studies with higher scores were randomised trials and they were judged to be appropriate in their statistical analyses and study designs. The risk of bias assessment is detailed in Table 2.

**Table 2:** Quality assessment outcome using the QuADS tool

Study (Year)	Theory/ Concept	Aims	Setting	Study design	Sampling	Data collection tools choice rationale	Tool format and content	Procedure description	Recruitment data	Analysis justification	Analysis appropriate	Stakeholders considered	Strengths and limitations	Total score	% score
Azman (2021) [19]	1	3	2	3	3	2	3	3	3	2	3	2	1	31	79
Berland (2019) [23]	1	3	2	2	2	3	3	3	2	3	3	2	2	31	79
Brewer (2021) [30]	1	3	3	3	2	3	3	3	3	3	3	2	3	35	90
Cho (2022) [18]	1	3	2	3	3	2	3	3	2	2	3	1	2	30	77
Somera (2021) [41]	1	3	3	3	2	3	3	3	2	3	3	0	2	31	79
Durán-Guerrero (2019) [16]	1	2	3	2	2	0	0	2	2	3	3	2	1	23	59
Enoch (2022) [33]	3	3	3	3	2	3	3	3	2	3	3	2	2	35	90
Flatt (2023) [35]	1	3	3	3	2	3	3	3	2	3	3	2	3	34	87
Gong (2021) [38]	1	1	3	2	2	3	3	2	1	3	3	2	2	28	72
Grosser (2019) [20]	1	3	3	3	2	2	3	3	3	3	3	2	2	33	85
Hansen (2020) [24]	1	2	3	2	2	0	3	3	2	3	2	2	2	27	69
Heriwardito (2023) [40]	1	3	3	3	2	2	3	3	3	3	3	0	2	31	79
Herrmann-Werner (2019) [22]	1	3	3	3	3	2	3	3	2	2	3	2	2	32	82
Huang (2022) [34]	3	3	3	2	3	3	3	3	2	3	3	2	2	35	90
Kasai (2021) [29]	1	3	3	2	2	2	3	3	2	3	3	2	2	31	79
Lang (2023) [36]	2	3	3	3	3	3	3	3	2	0	2	2	2	31	79
Lehmann (2019) [21]	2	3	3	3	3	2	2	3	2	3	3	2	2	33	85
Nathan (2022) [17]	1	3	3	3	3	3	3	3	3	2	3	2	3	35	90
Nazari (2020) [25]	2	3	3	3	2	3	3	3	2	3	3	2	2	34	87
Plackett (2020) [27]	2	3	2	3	2	3	3	3	3	3	3	2	2	34	87
Power (2020) [26]	1	2	3	2	2	3	3	3	2	0	0	2	2	25	64
Rajendran (2021) [37]	2	2	2	2	2	3	3	2	2	0	0	2	0	22	56
Saeed (2023a) [39]	3	3	3	2	2	2	3	3	2	0	2	2	3	30	77
Saeed (2023b) [42]	2	3	3	2	2	3	2	3	2	3	2	0	2	29	74
Viljoen (2020) [28]	1	3	3	2	3	3	3	3	2	3	3	2	2	33	85
Vincent (2022) [31]	2	3	3	3	2	3	3	3	2	3	3	2	2	34	87
Zaghal (2022) [32]	1	3	2	3	2	3	3	3	2	3	3	0	3	31	79

The full description of each criterion is detailed in Supplement 2. QuADS: Quality assessment with diverse studies tool

## Discussion

This systematic review highlights the growing role and potential of digital learning interventions in medical education. The broad geographical distribution and diverse study designs of the reviewed studies underscores the widespread interest and applicability of digital tools in enhancing clinical knowledge and skills. The study findings suggest that online learning platforms can be a valuable complement to traditional clinical education, offering flexible, engaging, and potentially more cost-effective training options.

Our analysis demonstrated that online modules and courses may improve exam scores and knowledge retention, particularly when compared to traditional lecture-based methods. This aligns with previous studies that have highlighted the benefits of e-learning in medical education, particularly in providing flexible, scalable, and accessible learning opportunities [11, 12]. The effectiveness of these tools in improving specific skills, such as diagnostic imaging [16] and cardiac auscultation [26], underscores their potential to supplement or even replace traditional teaching methods in certain contexts. Furthermore, virtual training in practical skills like suturing and knot-tying proved to be as effective as face-to-face training, though computer-based learning fell short in critical areas such as CPR training. This indicates that while digital interventions can be highly effective, they must be carefully matched to the skills being taught.

The results suggest that digital and blended learning interventions lead to sustained improvements in student performance and knowledge retention. Studies by Nathan et al. [17] and Azman et al. [19] showed significant gains in suturing skills and diagnostic abilities, consistent with literature suggesting that interactive and multimedia-enhanced learning can lead to better retention and application of knowledge [43, 44]. The findings from Kasai et al. [29] and Huang et al. [34] further support the notion that digital tools can enhance competency in various clinical tasks, although a balanced integration with traditional methods is often preferred.

High levels of student satisfaction and engagement with digital learning approaches were evident across multiple studies [20, 34, 39]. The studies reported that interactive and video-based learning environments were well-received by students, enhancing their engagement and satisfaction. This is in line with research that highlights the importance of interactivity and multimedia in maintaining student interest and motivation [45, 46], although the review by Ulum was not specific to medical education [45]. However, some studies noted that while digital methods were effective, students still valued the personal interaction and hands-on experience provided by traditional face-to-face training [31, 39]. The integration of multimedia resources, such as video-based learning, showed significant benefits in terms of engagement and comprehension, particularly in clinical contexts, though anatomical knowledge gains were less pronounced [20]. The success of blended learning approaches and virtual microscopy further supports the advantage of combining traditional and digital methods for optimal learning outcomes.

However, our review also identified a large amount of statistical and individual variability in the effectiveness of different digital interventions, with high dropout rates posing a challenge in some studies. Despite these challenges, the overall satisfaction and engagement levels were higher with digital learning modalities, suggesting that they can enhance the learning experience significantly.

Moreover, the potential cost savings associated with digital learning, as evidenced by the studies on suturing skills [17], present a compelling case for their broader implementation, especially in resource-limited settings. This finding is particularly relevant in the context of the COVID-19 pandemic, which necessitated rapid shifts to online learning and highlighted the need for cost-effective, scalable educational solutions [47].

### Implications for practice

The integration of digital and blended learning into medical education has profound implications for the future of the field. The adaptability and scalability of these tools can address the challenges posed by increasing student numbers and limited clinical training opportunities [11]. Furthermore, the ability to provide consistent and standardised training through digital platforms can potentially enhance the overall quality of medical education, ensuring that all students receive comprehensive and equitable training.

However, the preference for traditional methods in certain scenarios, as noted in the study by Huang et al. [34], suggests that a hybrid approach may be most effective. Combining the strengths of digital tools with the hands-on, interactive nature of traditional training can create a more holistic and effective educational experience. This hybrid model can leverage the flexibility and accessibility of digital learning while preserving the essential elements of face-to-face interaction and practical skill development.

Future research should focus on longitudinal studies to assess the long-term impact of digital and blended learning on clinical skills and professional competencies. Exploring the optimal balance between digital and traditional methods and understanding the factors influencing student preferences and learning outcomes, will be critical in shaping the future of medical education.

### Strengths and limitations

Strengths of our review included the diverse range of studies from various countries and studied interventions. This diversity enhances the generalisability of the findings across different educational contexts and healthcare systems. Additionally, the use of rigorous inclusion criteria and detailed risk of bias assessment, using the QuADS tool, ensures the quality and reliability of the synthesized evidence. However, the review also has limitations. The heterogeneity of the included studies, in terms of interventions, outcomes, and assessment methods, complicated direct comparisons and synthesis of results. The variability in study quality, as indicated by the wide range of QuADS tool scores, suggests that some findings should be interpreted with caution. Additionally, high dropout rates in several studies may have introduced bias, affecting the robustness of the conclusions. The lack of detailed cost analyses in most studies limits the ability to fully assess the economic impact of digital learning interventions. Despite these limitations, the review provides a comprehensive overview of the current landscape of digital learning in medical education and identifies key areas for future research and improvement.

## Conclusions

The findings of our review suggest that digital and blended learning methodologies may offer benefits in medical education, particularly in terms of knowledge acquisition, confidence building, and engagement. The effectiveness of these approaches varies depending on the skill being taught and the specific educational context. The integration of traditional and innovative teaching methods appears to offer the most comprehensive learning experience, underscoring the importance of a multifaceted approach to medical education. Future studies should look at the relative efficacy of the different digital modalities.

## Supplementary Information

Supplementary Material 1.

Supplementary Material 2.

## Data Availability

Data is provided within the manuscript or supplementary information files.

## Abbreviations

QuADS: Quality Assessment With Diverse Studies
PRISMA: Preferred Reporting Items for Systematic Reviews and Meta-Analyses
