# Supplementary material for: Digital learning of clinical skills and its impact on medical students’ academic performance: a systematic review
Source: BMC Med Educ. 2024 Dec 18;24:1477. doi: 10.1186/s12909-024-06471-2 (PMC11653901; doi:10.1186/s12909-024-06471-2)
Supplement: Supplementary file 1 — Supplementary Material 1. [file 12909_2024_6471_MOESM1_ESM.docx]

## Supplement 1: Search strategy

Each search was run in October 2023 and then updated on 1^st^ April 2024.

Search terms were adapted for each database using the polyglot search tool: https://polyglot.sr-accelerator.com/

**Search terms:**

clinical competence OR clinical competencies OR clinical competency OR clinical skill OR clinical skills OR competence, clinical OR competencies, clinical OR competency, clinical OR skill, clinical OR skills, clinical OR preceptorship OR Clinical placement OR academic performance OR academic performances OR academic test performance OR academic test performances OR academic test score OR academic test scores OR educational test performance OR educational test performances OR educational test score OR educational test scores OR performance, academic OR performance, academic test OR performance, educational test OR performances, academic OR performances, educational test OR score, academic test OR score, educational test OR scores, educational test OR test performance, academic OR test performance, educational OR test performances, educational OR test score, educational OR test scores, academic

AND

Medical Education OR medical educat* OR medical train* OR medical field training OR medical school* OR medical student* OR medical student OR student, medical OR students, medical

AND

distance learning OR education, distance OR education, online OR learning, distance OR learning, online OR online education OR online educations OR online learning OR Blended learning OR hybrid learning
